# The outdoor office: a pilot study of environmental qualities, experiences of office workers, and work-related well-being

**DOI:** 10.3389/fpsyg.2023.1214338

**Published:** 2023-12-07

**Authors:** Carina Söderlund, Luis Alfonso de la Fuente Suárez, Annika Tillander, Susanna Toivanen, Katarina Bälter

**Affiliations:** ^1^School of Innovation, Design and Engineering, the Division of Information Design, Mälardalen University, Västerås, Sweden; ^2^School of Architecture, Autonomous University of Nuevo Leon, San Nicolás de los Garza, Mexico; ^3^The Division of Statistics and Machine Learning, Department of Computer and Information Science, Linköping University, Linköping, Östergötland, Sweden; ^4^School of Health and Welfare, Mälardalen University, Västerås, Sweden; ^5^Department of Medical Epidemiology and Biostatistics, Karolinska Institute, Stockholm, Sweden

**Keywords:** outdoor office work, environmental experiences/perceptions, new ways of working, outdoor office space, work-related well being, visual and spatial information

## Abstract

Outdoor office work is an emerging aspect of the concept of ‘new ways of working’, but only sparse data are available about the environmental qualities of the outdoor office space, experiences of office workers, and work-related well-being of outdoor office work. Here, we present an exploratory pilot study on well-being and outdoor office work in a public urban space. An outdoor office was set up in the courtyard of a university campus, and the participants (*n* = 16) conducted office work outdoors for 30 min and thereafter participated in an eye-tracking session for 11–15 min (*n* = 8) and subsequently filled out surveys (*n* = 16). The eye tracker allowed the discovery of natural and built elements in the outdoor environment that caught the participants’ visual attention, whereas the surveys assessed aspects of their subjective experiences of the outdoor office space (its visual and spatial qualities) and the work there. The results are presented as network graphs where correlations are shown regarding different aspects of office work outdoors. The results indicate that outdoor office work in a public urban space may promote work-related well-being in terms of positive outdoor office space experiences. Based on the findings, a preliminary set of outdoor office qualities is proposed. Those qualities relate to the legibility and imageability of the outdoor office space, its focal points, and depth/spaciousness, in addition to attributes of usability and environmental richness, including if the outdoor office space affords natural contact and supports activities, in addition to social and individual interactions and relations.

## 1 Introduction

In recent decades, the designated built office has been challenged by the concept of ‘new ways of working’ (Appel-Meulenbroek, 2016), which is centered around flexible and connected employees who can work from anywhere at any time, supported by (digital) technologies (Ng, 2016; Kingma, 2019). Usually, office work is considered an indoor activity. However, Toivanen (2019) questions the ‘indoor norm’ and concludes that more than 50% of those conducting office-like tasks would like to work partly outdoors if it would be accepted by the organization.

Health and well-being benefits are major reasons for outdoor office work (Petersson Troije et al., 2021). This assumption is based on extensive research regarding health and well-being due to exposure to natural and urban green spaces, as summarized in a literature review by Nejade et al. (2022) and the World Health Organization (Egorov et al., 2016). Two well-established theories are the stress recovery theory on reduced physiological stress due to nature exposure (Ulrich et al., 1991) and the attention restoration theory on cognitive fatigue and restoration in green environments, which are well covered in the research reviews by Ohly et al. (2016) and Stevenson et al. (2018).

The research overview by Sadick and Kamardeen (2020) emphasizes that well-being in a broad sense among knowledge workers (office work is included) can be associated with their access to green environments. It applies to green environments indoors (Gritzka et al., 2020; Colenberg et al., 2021) via windows (Gilchrist et al., 2015) and outdoors (Lottrup et al., 2013; Gilchrist et al., 2015; Colley et al., 2017; Korpela et al., 2017; Cinderby and Bagwell, 2018; Hyvönen et al., 2018; Toivanen, 2018; Gritzka et al., 2020).

Nevertheless, workers’ access to green environments does not imply working in the open air in contrast to outdoor office work where being outdoors is the core component. In this context, ‘outdoor office’ is a relevant concept, as an extension/a spin-off of the designated built office (Söderlund et al., 2022). There is no formal definition; thus, we suggest the following: The outdoor office is (1) a formally organized or a partly deliberated/arranged space and fixed regarding location and facilities, or mobile when the location changes and the facilities are moved or (2) a free space regarding formalization, spatial organization, and location, merely identified by people being on-site and their impromptu office work activities.

Although research on outdoor office work is scarce, there are some previous studies, see, e.g., Lottrup et al. (2012), Mangone et al. (2017), Toivanen (2018), and Petersson Troije et al. (2021). Mangone et al. (2017) concluded that outdoor environments are suitable for work activities that overlap with office work. According to Petersson Troije et al. (2021), the external and physical outdoor environment, along with sociocultural and organizational aspects, are essential for outdoor office work and influence how easily and frequently office work will be performed outdoors. Moreover, the authors conclude that the outdoor office should be a calm and green place, close to the designated workplace, with facilities for digital work, among other conveniences. This relates to ideas by Lottrup et al. (2012), who build on Grahn and Stigsdotter’s (2010) notions of perceived sensory dimensions of urban green space. Office-related tasks in a serene undisturbed environment with few people and no littering are then strongly associated with reduced stress.

It is widely accepted that natural environments are more well-being-promoting than urban environments; however, there are indications that urban environment could have well-being benefits (Berto, 2014; Stigsdotter et al., 2017; Menardo et al., 2021), for instance, on psychological restoration (Stigsdotter et al., 2017) and affective/cognitive appraisals (Bornioli et al., 2018). Considering that a large proportion of employees work in cities, it is reasonable to locate an outdoor office in urban green spaces, that according to Egorov et al. (2016) can be private, semi-private, residential, or public areas including green (e.g., trees), blue (e.g., ponds), and built elements (e.g., bridges/sculptures). Additionally, from a democratic viewpoint, public space is also a sensible location for an outdoor office. Such spaces are for the masses and difficult to deny people access to Johnson and Glover (2013).

Nevertheless, public urban spaces can be serene or boisterous, providing dissimilar conditions for outdoor office work. The space can be spacious, rich in vegetation, and isolated from traffic, or a space between buildings of sparse vegetation, close to traffic and walkways. The relationship between outdoor office space and work-related well-being may differ depending on these prerequisites.

While the concept of well-being is widely debated (*cf.* Ryan and Deci, 2001; Ruggeri et al., 2020), we used the hedonic approach to explore potential well-being benefits in terms of workers’ outdoor office space experiences. The study concerns those with office-like tasks partly or fully in their professions (i.e., office workers). The aim is to investigate (a) office workers’ positive and negative experiences of the outdoor office space and (b) the visual and spatial elements in that space capturing the workers’ visual attention. The study will contribute to preliminary hypotheses regarding the qualities of outdoor offices in public urban spaces, based on human–environment relations.

## 2 Methods for empirical data collection

**Figure 1:**
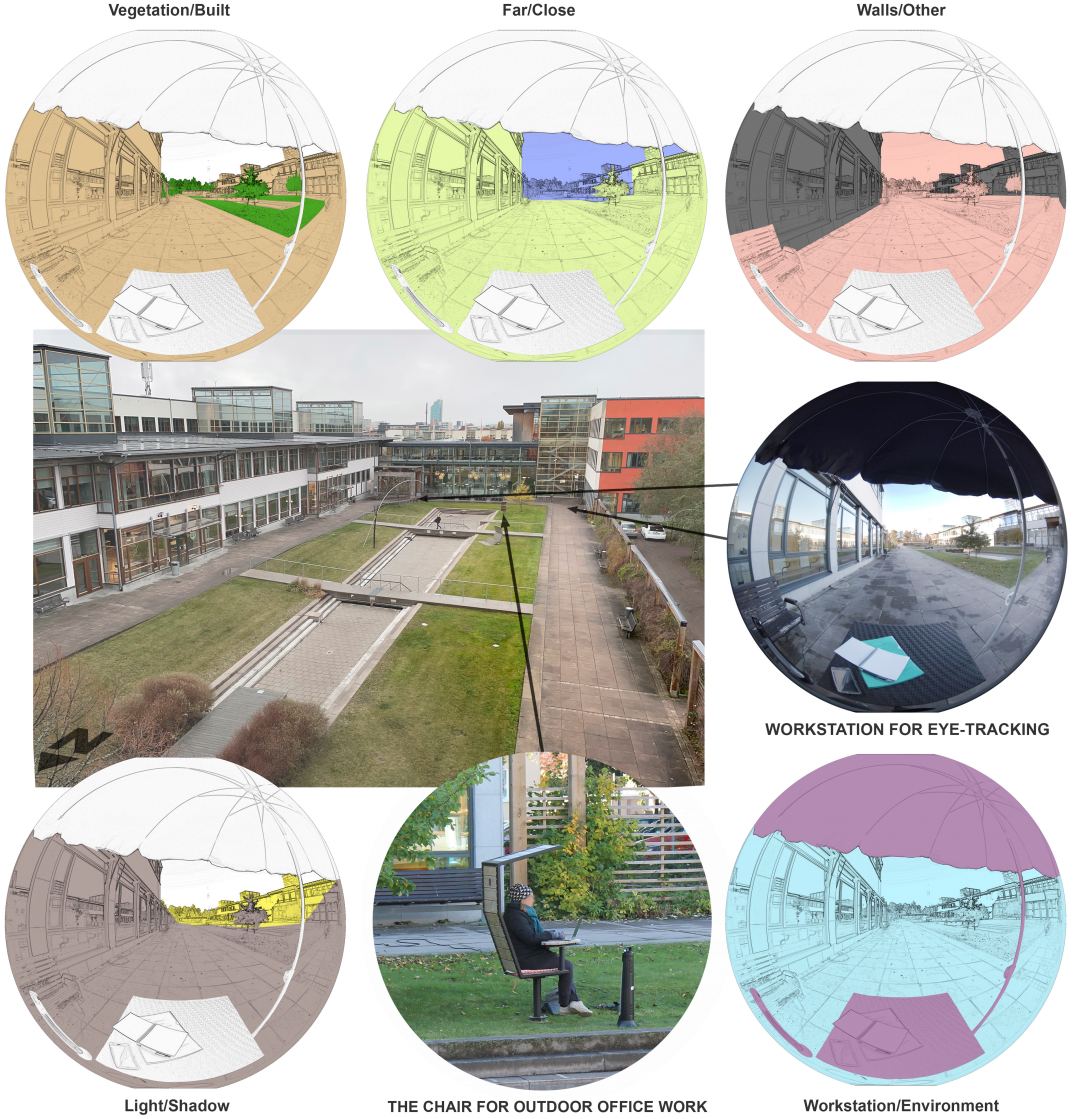
Explored outdoor office space and its areas of interest.

The outdoor office in this study was set up in the courtyard of a university campus in a Swedish city with a population of nearly 160 K (Statistics Sweden, 2023). A custom-designed fixed and rotatable office chair adjusted for outdoor work with an attached table and canopy, Figure 1, was installed on the lawn close to a pair of minor broad-leaved trees near a waterless concrete pond (≈60 by 6 m/197 by 20 ft), surrounded by patches of grass and a dozen broad-leaved trees in the far distance. This courtyard and the chair are part of a public space, partly enclosed by buildings in three directions with an open view to the northwest and close to parking lots, busy streets, and walkways. The university provided Wi-Fi, and electric cords and an infrared heater were installed next to the chair.

All employees (≈1000) at the campus were invited based on convenience sampling. To be included in the study, the participants were required to carry out office-like tasks during part of their workday, whether teaching, administration, or research. In total, 22 people responded and 16 participated in the study (11 female participants, and 5 male participants).

The study was conducted in the chair and the courtyard, i.e., the outdoor office space. The participants performed office work in the chair for 30 min since research specifies that such short time spent outdoors has positive health effects (e.g., Ryan et al., 2010). They then moved approximately 10–20 m (32–65 ft) to a shadier part for an eye-tracking session (11–15 min) as the daylight at the chair was too intense for the eye-tracking device. Next, on a nearby bench, a modified think-aloud technique was used, whereby the participants were observed when verbalizing their thoughts about the outdoor office space and the work there. Finally, in the vicinity, the participants completed a survey for approximately 15 min with the outdoor office within sight. The weather conditions were documented for each test session using photographs and measures of daylight intensity, temperature, and wind.

To gain ecological validity, the study was carried out during regular office hours from late September to mid-October and during typical weather conditions for the country with temperatures between 10–15°C (50–59°F). The study was conducted during both sunny and cloudy days with drizzle, wind, and wind gusts of 5–6 m/s (16.4–19.6 ft./s) and 9–11 m/s (29.5–36 ft./s). The participants were instructed to dress according to the weather.

In the study, only eye-tracking and survey data are presented.

### 2.1 Eye-tracking and areas of interest

After 30 min of office work, the participants used eye-tracking glasses to study what natural and built elements of the outdoor environment captured their visual attention since observing such elements could relate to the participants’ subjective experiences of the outdoor space. In short, visual attention refers to physiological and cognitive mechanisms allowing the reduction and selection of environmental stimuli, which is essential when identifying and recognizing objects in the environment (Evans et al., 2011). The participants were informed in advance that if they had any known eye problems or wore glasses, they could opt out of the eye-tracking activity. The participants were seated in a workstation (office chair and table) with a custom-made parasol providing protection from daylight and keeping the light level below 1,500 lux. The eye-tracking glasses (Tobi Pro Glasses 2, 50 Hz) were used under the supervision of a researcher. The study focused on observation duration, which the glasses recorded to discover what elements in the outdoor office space attracted the participants’ visual attention.

Thirteen (13) participants opted to take part in eye-tracking sessions, carried out during afternoons (2-4 p.m.), to have similar light and shadow conditions. The participants were instructed to stay seated for 15 min and reminded that the glasses recorded the outdoor space and sounds. Office work includes periods with and without focusing on office-like tasks. The eye-tracker first recorded what the participants observed while answering six general questions in writing about their usual workplace, such as the number of employees and the year of incorporation. Then, they were free from set tasks and remained seated, still wearing the glasses.

This study includes data from 8 participants as data from five participants were excluded due to recording issues and invalid results. Data from the eye-tracking glasses were analyzed with Tobii Pro Lab software (2021/1.181) with achieved gaze samples of 73–94%, with only one below 80%. A 360° photograph of the outdoor office space, as seen from the workstation, served as a basis for manually mapping the participants’ eye fixations. Thereafter, a set of areas of interest were identified and coupled in opposing pairs as follows:

- Vegetation in the outdoor environment versus built elements.
- Elements far from the workstation versus close elements.
- Daylight-illuminated elements in the environment versus elements in shadow.
- Building walls enclosing the outdoor environment versus the other elements.
- The overall outdoor environment versus the workstation (e.g., worktable and umbrella).

The observation duration ratio between these opposing pairs was calculated by dividing the first area of interest by the second. Five eye-tracking metrics were created and named ‘vegetation/built,’ ‘light/shadow,’ ‘walls/other,’ ‘far/close,’ and ‘environment/workstation’ (Figure 1).

### 2.2 The survey

All participants (*n* = 16) completed a survey to assess positive and negative experiences of the outdoor office space and the work there. The survey was developed with a Likert scale from 1 to 5, corresponding to, (1) very slightly or not at all, (2) a little, (3) moderately, (4) quite a bit, and (5) extremely (Supplementary Table S1). The survey’s statements reflect findings in recent outdoor office research and classical theories on urban experiences and architectural and environmental psychology. These statements were grouped accordingly:

- The cognitive and emotional aspects of working outdoors (CEW); 10 statements based on aspects of outdoor office work by Petersson Troije et al. (2021). For example: When I work in this outdoor office space ‘I feel good’/‘I can concentrate’/‘I feel stressed.’
- The outdoor office’s visual and spatial elements and their organization as perceived by the office workers (VSO); eight statements based on Lynch’s notions regarding the legibility of urban spaces (Lynch, 1960), including statements on spatial and visual information in urban green spaces (Kaplan and Kaplan, 1989; Kaplan et al., 1998). For example, I experience this outdoor office space as ‘a central point’/‘clearly defined’/‘has a clear structure.’
- The experience of the functions/purposes and actions related to the outdoor office space (FPA); 14 statements reflecting Lynch’s evolving ideas on city forms (Lynch 1984/1981), e.g., I experience this outdoor office space as ‘safe’/‘easy to change’/‘private.’
- Relations and activities related to public urban spaces (RAS); four statements inspired by Gehl’s (2011/1971), namely, I think this office space supports ‘work activities’, ‘social interactions’ and ‘close relations.’
- The positive/negative appeal of the outdoor office space (PNA); 38 statements reflecting Hesselgren’s positive and negative adjectives were also tested for estimating outdoor space experiences, as cited in Hesselgren et al. (1975). For example, this office place feels ‘neglected’/‘maintained’ (the adjectives were evaluated separately instead of employing a semantic differential scale).

### 2.3 Data processing procedure

**Figure 2:**
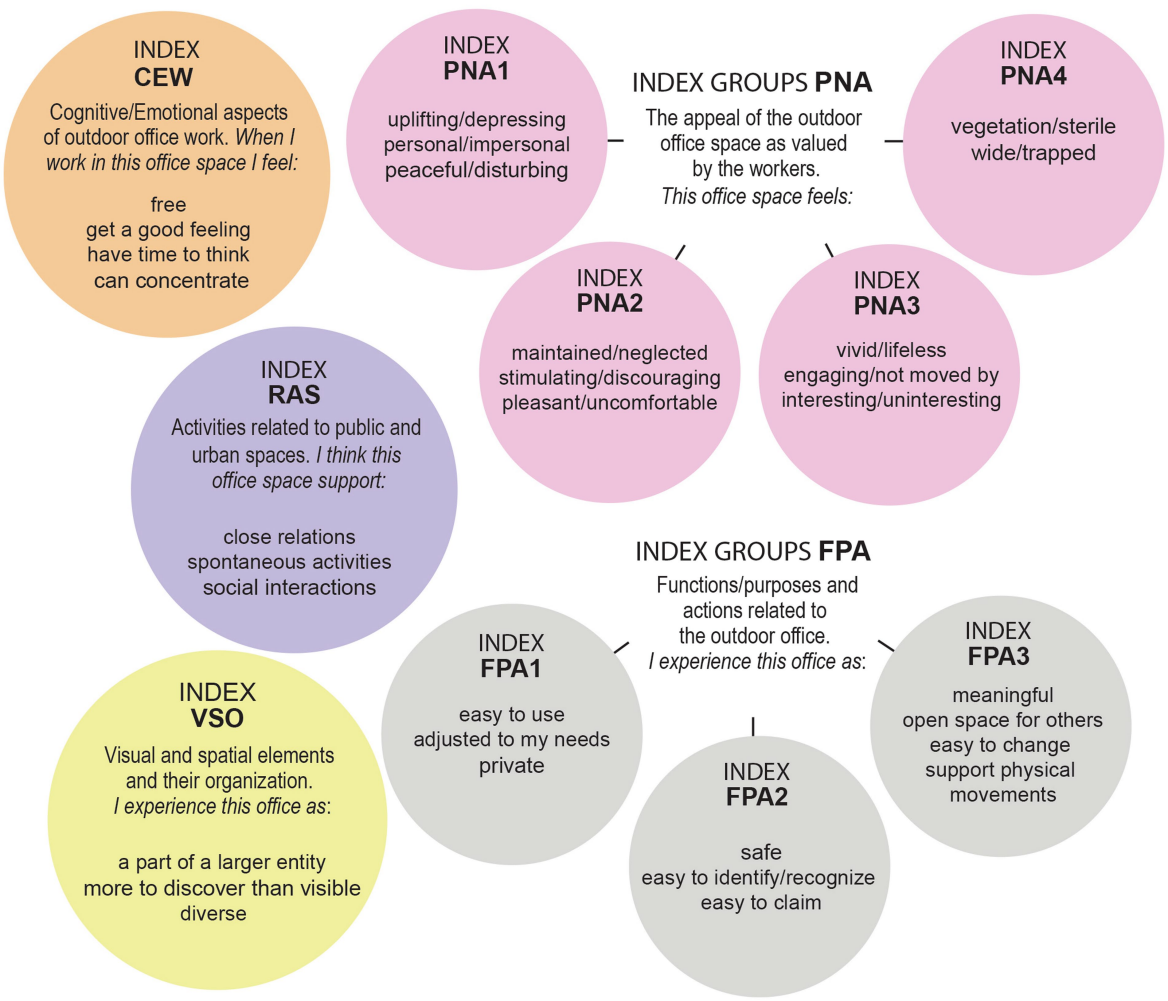
Ten identified indexes from survey data (*n* = 16), four indexes based on statements in FPA, three indexes with PNA statements, and one index each for the groups VSO, RAS, and CEW.

**Figure 3:**
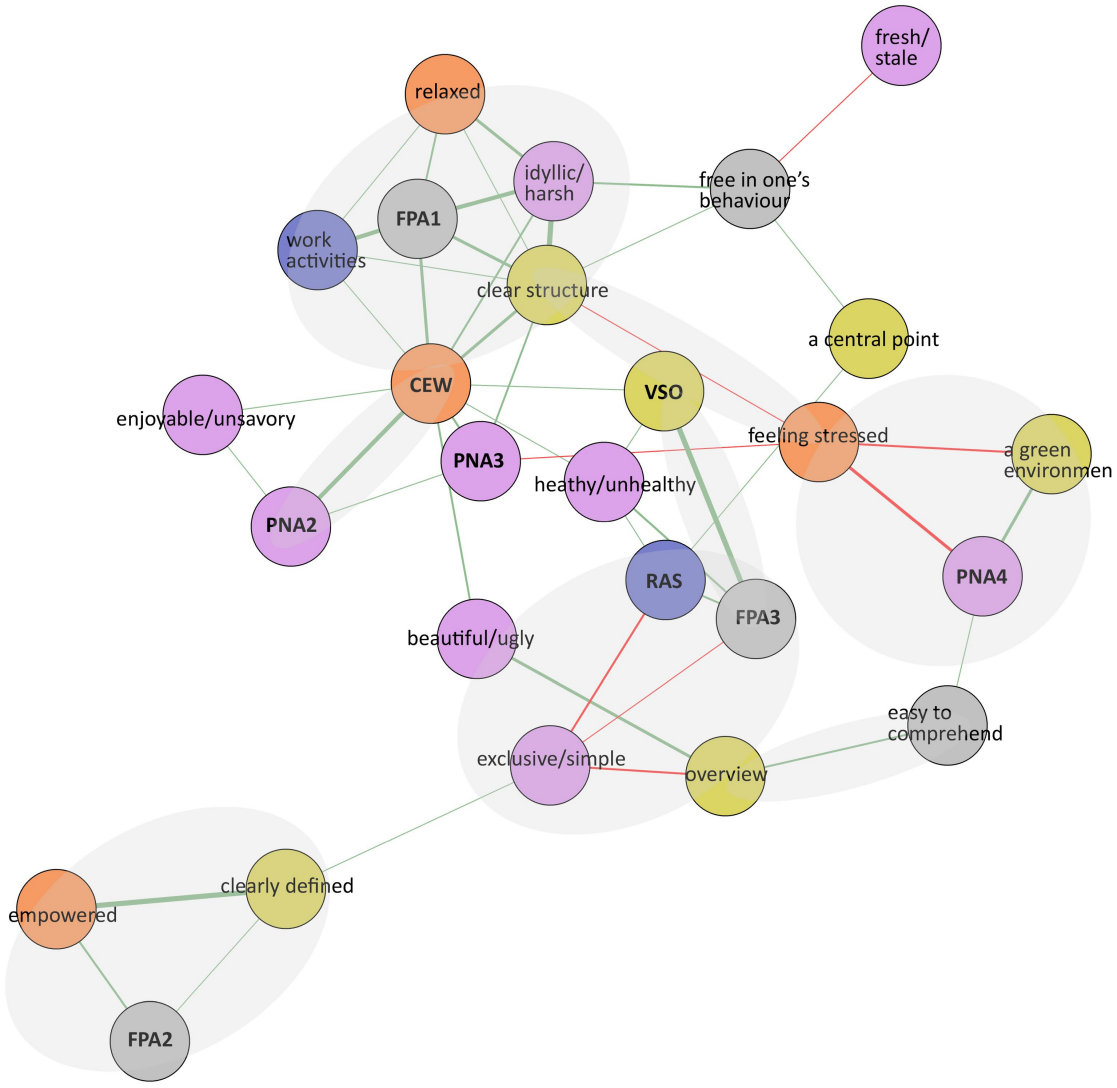
Network graph based on survey data (*n* = 16).

**Figure 4:**
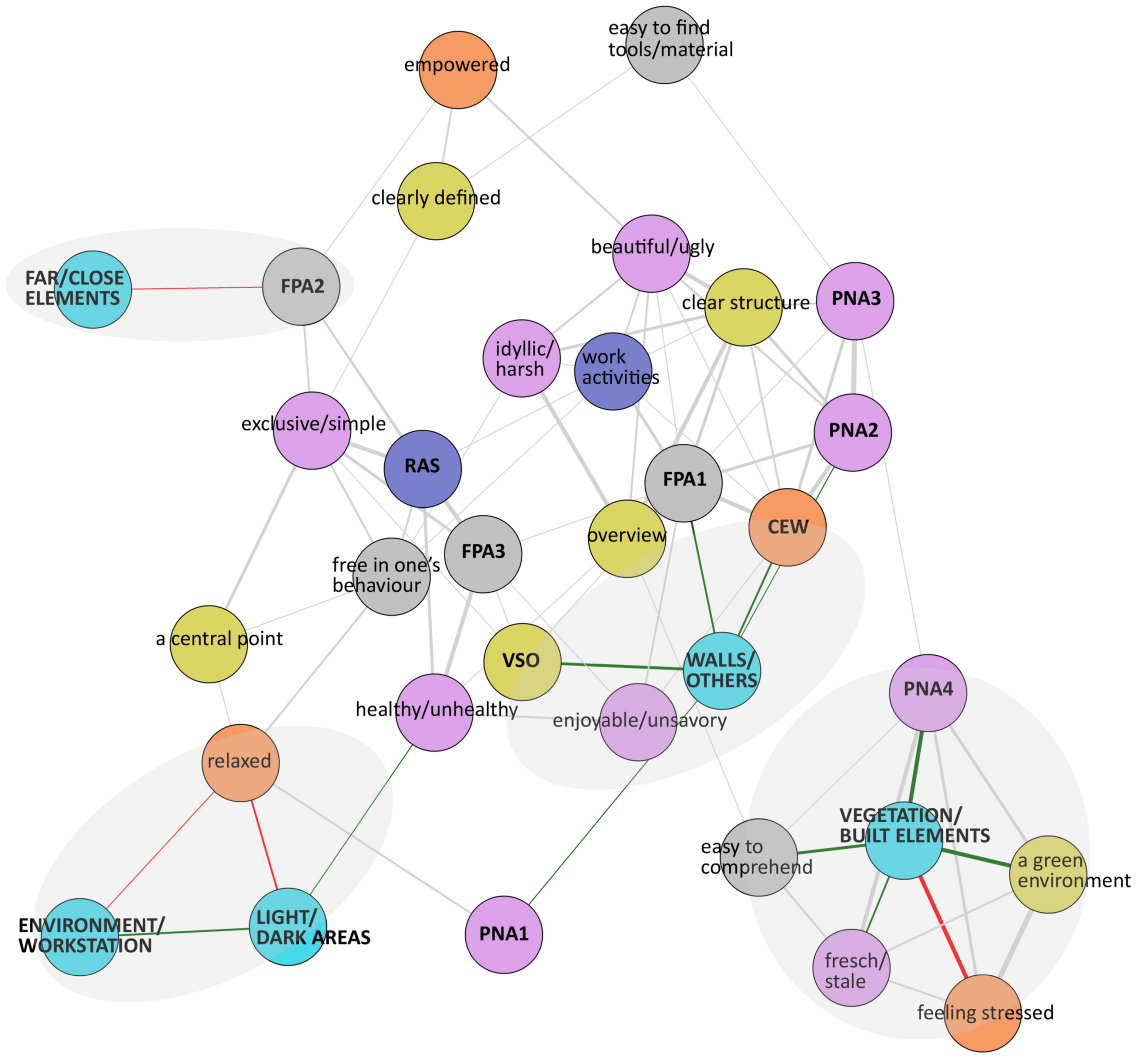
Network graph based on survey data and eye-tracking data (*n* = 8). The eye-tracking metrics are in bold text: vegetation/built, light/shadow, walls/others, far/close, and environment/workstation.

Survey and eye-tracking data were analyzed separately as follows, and then, merged in the final step:

1. Based on survey data (*n* = 16), statements with less than 20% unique responses were excluded and the statement ‘dejected’ was excluded due to low variation among the responses on the Likert scale of 1–5. The statements ‘silent/noisy and ‘smelly/fragrant’ were excluded as the study focuses on visual experiences. ‘Not at work’ was excluded due to a misspelling. Data were missing once regarding the statements ‘maintained’ and ‘sterile’, respectively, and imputed with the value 0.
2. For each statement in the PNA group (e.g., the adjectives neglected/maintained), we calculated a combined overall adjective for each paired statement as the difference between the response for the positive adjective and the response for the negative adjective. A combined adjective ranged between the scores −1 to −4 (negative), 0 (neutral), and 1–4 (positive).
3. Spearman’s rank correlation (*r*) was used to measure the pairwise relation between all statements within each group (CEW/VSO/FPA/RAS/PNA). For example, the statement ‘I feel free’ was correlated with all other statements in group CEW, and so on. Correlated statements with a cutoff value above *r* = 0.5/below *r* = −0.5 were plotted in a network graph, and groups were identified to create 10 indexes; 4 indexes based on statements in FPA, 3 indexes with PNA statements, and 1 index each for the groups VSO, RAS, and CEW (see Figure 2). Statements without a strong correlation to the respective index were considered stand-alone statements.
4. The Spearman’s rank correlation between the 10 indexes and stand-alone statements was calculated. Correlations with a cutoff of *r* = 0.5 and above are presented in a network graph (see Figure 3).
5. Spearman’s rank correlation (*r*) was used to calculate the relation between the subset with survey data (*n* = 16) and the eye-tracking metrics (*n* = 8). The result is presented in a network graph with a cutoff of *r* = 0.63 (Figure 4).

The analyses were conducted in R version 4.2.2 and the package “igraph” was used for displaying the rank correlation-based networks.

## 3 Results

Two network graphs present correlations regarding survey data (*n* = 16; Figure 3), and eye-tracking and survey data (*n* = 8; Figure 4). In the graphs, positive and negative correlations are green and red. The linewidth represents the strength of the correlation. The light-gray shapes were added afterward to highlight associations of importance based on the correlation strength, in addition to the number of indexes and statements linked to each other, to illustrate a comprehensive narrative (see explanations of the graphs’ abbreviations in Supplementary Table S2).

Figure 3 presents a cluster of positive correlations of the indexes CEW, FPA1, and the statements ‘clear structure’ and ‘idyllic/harsh.’ This indicates that the more clearly structured the outdoor office space was considered, the more idyllic, private, adjusted to the participants’ needs, and easy-to-use was perceived. These correlations positively relate to the participants’ perception of concentration, sense of freedom, having time to think, and a general good feeling when working outdoors.

Index FPA1 is also positively correlated to the statements ‘relaxed’ and ‘idyllic/harsh.’ This indicates that the more private, easier to use, and adjusted to the need office space was considered, the more idyllic it was perceived, and the more relaxed the participants felt when working. Additionally, the index FPA1 is positively correlated to the statements ‘work activities’ and ‘clear structure.’ This indicates, that the more private, easier to use, and adjusted to needs the outdoor office was perceived, the more structured it was seen and the more it was considered to support the work activities.

The statements ‘clear structure’ and ‘stress’ are negatively correlated, indicating that the clearer the structure of the outdoor office space, the less stress the participants experienced. Additionally, the statement ‘stressed’ is negatively correlated to ‘green environment,’ and index PNA4, implying that the more the office space was experienced as wide and a green area rich in vegetation, was related to less stress among the participants.

Index FPA2 is positively correlated to the statements ‘empowered’ and ‘clearly defined.’ It indicates that the more the outdoor office space was seen as easy to claim, identify/recognize, and safe, the more clearly defined it was perceived, and the more empowered the participants considered to feel when working there.

The indexes RAS and FPA3 are positively correlated, and both are negatively correlated with the statement ‘exclusive/simple.’ This implies that the more the outdoor office was seen as supportive of close relations, spontaneous activities, and social interaction, the more the space was seen as meaningful, easy to change, a space open for others, including supportive of physical movement, in addition, the less exclusive the participants experienced the space.

The indexes VSO and FPA3 are positively correlated. It indicates that the more the outdoor office space was considered diverse, part of a larger entity, and provided more to discover than first visible, the more meaningful, open for others, easy to change, and supportive of physical movements the space was perceived. The statement ‘easy to overview’ is positively correlated with both ‘easy to comprehend’ and ‘beautiful/ugly’ implying the easier the outdoor office space was to overview, the easier it was to comprehend and the more beautiful it was considered.

In Figure 4, the eye-tracking metric ‘vegetation/built’ positively correlates with the statements ‘easy to comprehend,’ ‘green environment,’ ‘fresh/stale,’ and the index PNA4. This indicates that the more the participants observed the vegetation versus built elements in the outdoor environment, the more they considered the outdoor office as a green environment, easier to comprehend, fresher, wider, and richer in vegetation. Additionally, the metric ‘vegetation/built’ is negatively correlated to the statement ‘feeling stressed,’ indicating that the more the participants observed the vegetation compared to the built elements in the outdoor office, the less stress the participants perceived they were experiencing.

The metric ‘wall/other’ positively correlates with index VSO, implying that the more the participants observed the building walls versus the other elements in the outdoor environment, the more the outdoor office was considered diverse, part of a larger entity, and had more to discover than first visible. Additionally, the metric ‘wall/other’ positively correlates to the indexes FPA1 and CEW. This indicates that the more the walls were observed by the participants versus the other elements outdoors, the more private the outdoor office space was perceived, easier to use, and adjusted to their needs. Additionally, the higher the value of ‘wall/other,’ the more the participants considered feeling good and free, being able to concentrate, and having time to think.

The index FPA2 is negatively correlated to the metric ‘far/close.’ This indicates that the harder it was for the participants to recognize/identify, claim, and feel safe in the outdoor office, the more they observed distant areas compared with closer areas in the outdoor environment. The metrics ‘light/shadow’ and ‘environment/workstation’ are positively correlated, but both are negatively correlated to the statement ‘relaxed.’ This indicates that the less relaxed the participants were, the more they observed daylight-illuminated elements in the outdoor office space beside shadowed elements and the more they viewed the overall outdoor environment versus the workstation.

## 4 Discussion and conclusion

This exploratory pilot study investigated the relationship between an outdoor office in a public urban space and office workers’ well-being in terms of their positive and negative experiences of that space and their work there. The findings indicate that access to green elements/environments when conducting outdoor office work could positively affect well-being, as aligned with previous research (*cf.* Korpela et al., 2017; Hyvönen et al., 2018; Steve and Bagwell, 2018; Gritzka et al., 2020). Additionally, the findings highlight the structure/organization of the outdoor office space as it may reduce the experience of stress. More to see than immediately visible in the outdoor environment may contribute to the experience of having time to think and being able to concentrate when conducting outdoor office work. These assumptions pertain to theories on cognitive restoration and legibility of green environments, see Kaplan et al. (1998). The findings also relate to notions by Petersson Troije et al. (2021) regarding office workers’ sense of empowerment when working outdoors. Findings in the current study implied that this sense of empowerment could also be affected by the design and usability of the outdoor office space, as being safe, and easy to claim and identify/recognize, which in turn were positively correlated with the sense of relaxation.

Moreover, being able to overview (look over) the outdoor office space correlated with its comprehensibility and perceived beauty. This compares with previous findings on affective responses in relation to focal points and spaciousness/depth in the natural environment (Ulrich, 1983) and positive psychological effects of extensive views (de la Fuente Suárez and Martínez-Soto, 2022).

Furthermore, our study highlights the importance of privacy when working outdoors, which is emphasized by Lottrup et al. (2012) and Petersson Troije et al. (2021) in terms of serene and undisturbed (green) environments. Nevertheless, this study indicates that sensing privacy could also relate to the outdoor office design including how clearly defined, easy to use, and adjusted to the needs it is. In addition, the findings indicate that university building walls partly framing an outdoor office space bring positive experiences of concentration, time to think, and a sense of freedom and privacy, including the perception of a diverse environment with more to discover than first visible. The latter may appear contradictory; however, buildings indicate a space and (human) activities beyond the outdoor environment.

Furthermore, the findings highlight that an outdoor office space, which is open for others, besides supporting physical movements, social interactions, close relationships, and spontaneous activities could bring positive outdoor office experiences. This is of particular interest for outdoor offices in urban spaces since such spaces gain meaning from peoples’ interactions and activities taking place there, as expressed by Gehl (2011/1971).

Based on these findings, we tentatively suggest those as follows: (a) Office work in public urban spaces can promote work-related well-being in terms of positive outdoor office experiences; and (b) Such experiences can be synthesized by the following six outdoor office qualities (neither ranked nor weighted) referring to the physical outdoor environment and office workers’ perception of it when the outdoor office space is considered:

- Easy to overview, identify/recognize, comprehend, and clearly structured and defined (i.e., legibility and imageability).
- Diverse, part of a larger entity, with more to discover than first visible, and partly enclosed by building walls (i.e., environmental enrichment).
- Easy to claim, safe, easy-to-use, and adjustable to needs (i.e., usability attributes).
- Part of green environments and/or having elements of vegetation (i.e., affording natural contact).
- Offering daylight-illuminated elements and distant view/s (i.e., focal points and depth/spaciousness).
- Open for others, supporting social interactions, close relations, and spontaneous activities (i.e., individual activities and human interactivity).

### 4.1 Limitations and future research

This exploratory pilot study does not provide statistically significant results; nonetheless, the findings are hypothesis-generating. A major limitation is the risk of spurious correlation due to few participants responding to numerous statements and few valid eye-tracking results partly because of sunlight intensity variations. In this study, workers’ subjective experiences of an outdoor office and the work there were collected via a survey. The survey data were combined with eye-tracking data to explore the built and natural elements of the outdoor environment that could relate to the subjective experiences of the actual environment. Multiple data-collecting techniques based on subjective and objective data contributed to a comprehensive view of the outdoor office space and the human-environment relation. Future studies should aim at including more participants, with different attitudes and habits of outdoor (office) work, working during longer periods at different occasions and locations.

## Data availability statement

The datasets presented in this article are not readily available because anonymized data can be shared, but not identifiable data. Data can be shared as long as the sharing does not violate the ethical review carried out prior to the study and the existing agreements with the participants in the study. Requests to access the datasets should be directed to carina.soderlund@mdu.se.

## Ethics statement

The studies involving humans were approved by the ethics review authority DNR 0222-03717-01. The studies involving humans were approved by the Swedish Ethical Review Authority Dnr 0222-03717-0. The participants provided their written informed consent to participate in this study. Written informed consent was obtained from the individual(s) for the publication of any potentially identifiable images or data included in this article.

## Author contributions

CS and ST prepared the test environment and ethical approval application. CS performed the identification of the study, created the study design and methods, and carried out data collection. LF explored the areas of interest and performed eye-tracking metrics. CS and LF performed mapping of gaze data and did data analysis. AT and LF performed statistical reasoning and AT performed data statistics. All authors contributed to the writing of the article and approved the submitted version.

## References

[ref1] Appel-Meulenbroek R. (2016). Modern offices and new ways of working are studied in more detail. J. Corporate Real Estate 18, 2–3. doi: 10.1108/JCRE-02-2016-0010

[ref2] Berto R. (2014). The role of nature in coping with psycho-physiological stress: a literature review on restorativeness. Behav. Sci. 4, 394–409. doi: 10.3390/bs4040394, PMID: 25431444 PMC4287696

[ref3] Bornioli A. Parkhurst G. Morgan P. L. (2018). The psychological wellbeing benefits of place engagement during walking in urban environments: a qualitative photo-elicitation study. Health Place 53, 228–236. doi: 10.1016/j.healthplace.2018.08.018, PMID: 30195155

[ref1003] Cinderby S. Bagwell S. (2018). Exploring the co‐benefits of urban green infrastructure improvements for businesses and workers’ wellbeing. Area. 50, 126–135.

[ref4] Colenberg S. Jylhä T. Arkesteijn M. (2021). The relationship between interior office space and employee health and well-being–a literature review. Build. Res. Inf. 49, 352–366. doi: 10.1080/09613218.2019.1710098

[ref5] Colley K. Brown C. Montarzino A. (2017). Understanding knowledge workers’ interactions with workplace greenspace: open space use and restoration experiences at urban-fringe business sites. Environ. Behav. 49, 314–338. doi: 10.1177/0013916516629194

[ref6] de la Fuente Suárez L. A. Martínez-Soto J. (2022). Relaxation and fascination through outside views of Mexican dwellings. Architecture 2, 334–361. doi: 10.3390/architecture2020019

[ref7] Egorov A. I. Mudu P. Braubach M. Martuzzi M. (2016). Urban Green Spaces and Health – A Review of Evidence. Copenhagen: WHO Regional Office for Europe.

[ref8] Evans K. K. Horowitz T. S. Howe P. Pedersini R. Reijnen E. Pinto Y.. (2011). Visual attention. Wiley Interdiscip. Rev. Cogn. Sci. 2, 503–514. doi: 10.1002/wcs.127, PMID: 26302302

[ref9] Gehl J. (2011/1971). Life between Buildings: Using Public Space, Washington DC: Island Press [E-book]

[ref10] Gilchrist K. Brown C. Montarzino A. (2015). Workplace settings and wellbeing: greenspace use and views contribute to employee wellbeing at peri-urban business sites. Landsc. Urban Plan. 138, 32–40. doi: 10.1016/j.landurbplan.2015.02.004

[ref11] Grahn P. Stigsdotter U. K. (2010). The relation between perceived sensory dimensions of urban green space and stress restoration. Landsc. Urban Plan. 94, 264–275. doi: 10.1016/j.landurbplan.2009.10.012

[ref12] Gritzka S. MacIntyre T. E. Dörfel D. Baker-Blanc J. L. Calogiuri G. (2020). The effects of workplace nature-based interventions on the mental health and well-being of employees: a systematic review. Front. Psych. 11:323. doi: 10.3389/fpsyt.2020.00323, PMID: 32411026 PMC7198870

[ref13] Hesselgren S. Brodin C. Gärling T. Toomingas A. Sivik L. (1975). Arkitekturpsykologi. En översikt samt något som stadsmiljöupplevelse, rumsupplevelse och färgupplevelse, R24:1975. LiberTr: Statens Råd för Byggnadsforskning Stockholm.

[ref15] Hyvönen K. Törnroos K. Salonen K. Korpela K. Feldt T. Kinnunen U. (2018). Profiles of nature exposure and outdoor activities associated with occupational well-being among employees. Front. Psychol. 9:754. doi: 10.3389/fpsyg.2018.00754, PMID: 29867699 PMC5968374

[ref16] Johnson A. J. Glover T. D. (2013). Understanding urban public space in a leisure context. Leis. Sci. 35, 190–197. doi: 10.1080/01490400.2013.761922

[ref17] Kaplan R. Kaplan S., (1989). The Experience of Nature. New York: Cambridge University Press.

[ref18] Kaplan R. Kaplan S. Ryan R. (1998). With People in Mind: Design and Management of Everyday Nature. Washington, DC: Island press.

[ref19] Kingma S. (2019). New ways of working (NWW): work space and cultural change in virtualizing organizations. Cult. Organ. 25, 383–406. doi: 10.1080/14759551.2018.1427747

[ref20] Korpela K. De Bloom J. Sianoja M. Pasanen T. Kinnunen U. (2017). Nature at home and at work: naturally good? Links between window views, indoor plants, outdoor activities and employee well-being over one year. Landsc. Urban Plan. 160, 38–47. doi: 10.1016/j.landurbplan.2016.12.005

[ref21] Lottrup L. Grahn P. Stigsdotter U. K. (2013). Workplace greenery and perceived level of stress: benefits of access to a green outdoor environment at the workplace. Landsc. Urban Plan. 110, 5–11. doi: 10.1016/j.landurbplan.2012.09.002

[ref22] Lottrup L. Stigsdotter U. K. Meilby H. Corazon S. S. (2012). Associations between use, activities and characteristics of the outdoor environment at workplaces. Urban For. Urban Green. 11, 159–168. doi: 10.1016/j.ufug.2011.12.006

[ref23] Lynch K. (1960). The Image of the City. Cambridge, MA: MIT Press.

[ref24] Lynch K. (1984/1981). A Theory of Good City Form. Cambridge, MA: MIT Press.

[ref25] Mangone G. Capaldi C. A. van Allen Z. M. Luscuere P. G. (2017). Bringing nature to work: preferences and perceptions of constructed indoor and natural outdoor workspaces. Urban For. Urban Green. 23, 1–12. doi: 10.1016/j.ufug.2017.02.009

[ref26] Menardo E. Brondino M. Hall R. Pasini M. (2021). Restorativeness in natural and urban environments: a meta-analysis. Psychol. Rep. 124, 417–437. doi: 10.1177/0033294119884063 31694463

[ref27] Nejade R. M. Grace D. Bowman L. R. (2022). What is the impact of nature on human health? A scoping review of the literature. J. Glob. Health 12. doi: 10.7189/jogh.12.04099 PMC9754067 36520498

[ref28] Ng C. F. (2016). Public spaces as workplace for mobile knowledge workers. J. Corporate Real Estate 18, 209–223. doi: 10.1108/JCRE-10-2015-0030

[ref29] Ohly H. White M. P. Wheeler B. W. Bethel A. Ukoumunne O. C. Nikolaou V.. (2016). Attention restoration theory: a systematic review of the attention restoration potential of exposure to natural environments. J. Toxicol. Environ. Health B 19, 305–343. doi: 10.1080/10937404.2016.1196155, PMID: 27668460

[ref30] Petersson Troije C. Lisberg Jensen E. Stenfors C. Bodin Danielsson C. Hoff E. Mårtensson F.. (2021). Outdoor office work–an interactive research project showing the way out. Front. Psychol. 12:636091. doi: 10.3389/fpsyg.2021.636091, PMID: 33912111 PMC8072124

[ref31] Ruggeri K. Garcia-Garzon E. Maguire Á. Matz S. Huppert F. A. (2020). Well-being is more than happiness and life satisfaction: a multidimensional analysis of 21 countries. Health Qual. Life Outcomes 18, 1–16. doi: 10.1186/s12955-020-01423-y 32560725 PMC7304199

[ref1002] Ryan R. M. Deci E. L. (2001). On happiness and human potentials: A review of research on hedonic and eudaimonic well-being. Ann. Rev. Psychol. 52, 141–166. 11148302 10.1146/annurev.psych.52.1.141

[ref1004] Ryan R. M. Weinstein N. Bernstein J. Brown R. Mistretta L. Gagné M.. (2010). Vitalizing effects of being outdoors and in nature. J. Environ. Psychol. 30, 159–168., PMID: 26302302

[ref32] Sadick A. M. Kamardeen I. (2020). Enhancing employees’ performance and well-being with nature exposure embedded office workplace design. J Build Eng. 32:101789. doi: 10.1016/j.jobe.2020.101789

[ref33] Söderlund C. de la Fuente Suarez L. A. Toivanen S. (2022). Environmental Dimensions of the Outdoor Office–Related to Work Experiences and Well-Being. In Conference, FALF 2022, 13–15 June, Kiruna Framtidens Arbete–Arbetets Framtid (Work in the Future, the Future of Work) (pp. 14–16).

[ref34] Statistics Sweden. (2023). The future population of Sweden 2020-2030. Available at: https://www.urn:nbn:se:scb-2020-be18sm2002_pdf

[ref35] Stevenson M. P. Schilhab T. Bentsen P. (2018). Attention restoration theory II: a systematic review to clarify attention processes affected by exposure to natural environments. J. Toxicol. Environ. Health B 21, 227–268. doi: 10.1080/10937404.2018.1505571 30130463

[ref36] Stigsdotter C. Corazon S. S. Sidenius U. Kristiansen J. Grahn P. (2017). It is not all bad for the grey city – a crossover study on physiological and psychological restoration in a forest and an urban environment. Health Place 46, 145–154. doi: 10.1016/j.healthplace.2017.05.007, PMID: 28528275

[ref37] Toivanen S. (2018). “Arbetsmiljö och utformning av aktivitetsbaserade kontor” in Gränslöst Arbete. En Forskarantologi om Arbetsmiljöutmaningar i Anknytning till ett Gränslöst Arbetsliv. ed. Aronsson G. (Stockholm: Rapport 2018:1. Arbetsmiljöverket)

[ref38] Toivanen S. (2019). Ute är Inne -Kom Igång Med kontorsarbete Utomhus [Outdoors is the New Black -Get Started with Outdoors Office Work]. Stockholm, Sweden: Akademiska Hus.

[ref39] Ulrich R. S. (1983). “Aesthetic and affective response to the natural environment” in Behavior and the Natural Environment. eds. Altman I. Wohlwill F. J. (Boston, MA, USA: Springer), 85–125.

[ref40] Ulrich R. S. Simons R. F. Losito B. D. Fiorito E. Miles M. A. Zelson M. (1991). Stress recovery during exposure to natural and urban environments. J. Environ. Psychol. 11, 201–230. doi: 10.1016/S0272-4944(05)80184-7

