# Supplementary material for: The outdoor office: a pilot study of environmental qualities, experiences of office workers, and work-related well-being
Source: Front Psychol. 2023 Dec 7;14:1214338. doi: 10.3389/fpsyg.2023.1214338 (PMC10758605; doi:10.3389/fpsyg.2023.1214338)
Supplement: Supplementary file 1 [file Table_1.DOCX]

**Table 1.**

The table presents the statements and Likert scale that were applied in this study. The first statements, which are not included in the paper, concern positive or negative affects related to outdoor office space, based on the PANAS Scales. See Watson, D., Clark, L. A., & Tellegen, A. (1988). Development and validation of brief measures of positive and negative affect: the PANAS scales. *Journal of personality and social psychology*, *54*(6), 1063.

**A Scale for Estimating Outdoor Office Work and the Surrounding Environment**

1 Very slightly (or not at all)

2 A little

3 Moderately

4 Quite a bit

5 Extremely

| **When I work at this office space…** | | | |  |
| --- | --- | --- | --- | --- |
| I feel free |  | I get new ideas |  | |
| I can concentrate |  | I feel that I am empowered |  | |
| it feels like I’m not at work |  | I get time to think |  | |
| I get a good feeling |  | I feel stressed |  | |
| I feel relaxed |  | I feel dejected |  | |

**I experience this office space …**

| as it has a clear structure |  | safe (physically and socially) |  |  |
| --- | --- | --- | --- | --- |
| clearly defined |  | easy to identify/recognize |  |  |
| a central point |  | easy to claim (to make to my own) |  |  |
| easy to use |  | as a green environment (close to e.g., plants, grass, parks) |  |  |
| easy to gain an overview |  | more to discover than what is visible |  |  |
| adjusted to my needs |  | private |  |  |
| an open space for others |  | diverse |  |  |
| easy to comprehend |  | meaningful |  |  |
| inviting |  | easy to find the tools/material I seek for |  |  |
| easy to change |  | a part of a larger entity |  |  |
| **I think this office space supports…** | | | | |
|  | | | | |
| social interactions |  | to be free in one’s behaviour |  |  |
| close relations |  | work activities |  |  |
| spontaneous activities |  | move physically |  |  |

**This office space feels…**

| Neglected |  | Well maintained |  |
| --- | --- | --- | --- |
| Stale |  | Fresh |  |
| Interesting |  | Uninteresting |  |
| Harsh |  | Idyllic |  |
| Uncomfortable |  | Pleasant |  |
| Uplifting |  | Depressing |  |
| Simple |  | Exclusive |  |
| Disturbing |  | Peaceful |  |
| Noisy |  | Silent |  |
| Personal |  | Impersonal |  |
| Unhealthy |  | Healthy |  |
| Ugly |  | Beautiful |  |
| Unsavory |  | Enjoyable |  |
| Smelly |  | Fragrant |  |
| Not moved by |  | Engaging |  |
| Rich in vegetation |  | Sterile |  |
| Wide |  | Trapped |  |
| Vivid |  | Lifeless |  |
| Discourage |  | Stimulating |  |
