# Supplementary material for: The outdoor office: a pilot study of environmental qualities, experiences of office workers, and work-related well-being
Source: Front Psychol. 2023 Dec 7;14:1214338. doi: 10.3389/fpsyg.2023.1214338 (PMC10758605; doi:10.3389/fpsyg.2023.1214338)
Supplement: Supplementary file 2 [file Table_2.pdf]

Table 2. Abbreviated titles of the statements and metrics appearing in the correlation networks of Figures 3 and 4.

Group	Abbreviation	Statement(s)
CEW<br>Cognitive/emotional aspects of office work outdoors.<br><br>Color: orange<br><br>*When I work at this office space I feel…*	*empowered*	I feel that I am empowered.
	feeling stressed	I feel stressed.
	relaxed	I feel relaxed.
	CEW	I feel free, I get a good feeling, I have time to think, I can concentrate.
VSO<br>Visual and spatial elements and their organization.<br><br>Color: yellow<br><br>*I experience this office space…*	*clear structure*	as it has a clear structure.
		clearly defined.
		a central point.
		easy to comprehend.
	overview	easy to gain an overview.
	a green environment	as a green environment.
	VSO	A part of a larger entity, more to discover than what is visible, diverse.
FPA<br>Functions/purposes and actions related to the outdoor office space.<br><br>Color: grey<br><br>*I experience this office space…*	*free in one's behaviour*	to be free in one's behaviour.
	easy to find tools/ material	easy to find the tools/material you seek
		easy to comprehend
	FPA1	easy to use, adjusted on my needs, private.
	FPA2	safe, easy to identify/recognize, easy to claim.
	FPA3	Meaningful, an open space for others, easy to change,<br>*I think this office space supports…* to move physically.
RAS<br>Relations and activities related to public and urban spaces.<br><br>Color: violet<br><br>*I think this office space supports…*		work activities.
	RAS	close relations, spontaneous activities, social interactions.
PNA<br>The positive/negative appeal of the outdoor office space.<br><br>Color: pink<br><br>*This office place feels…*		fresh – stale.
		exclusive – simple.
		healthy – unhealthy.
		idyllic – harsh.
		enjoyable – unsavory.
		beautiful – ugly.
	PNA1	uplifting – depressing, personal – impersonal, peaceful – disturbing.
	PNA2	well maintained – neglected, stimulating – discouraging, pleasant – uncomfortable.
	PNA3	vivid – lifeless, engaging – not moved by, interesting – uninteresting.
	PNA4	rich in vegetation – sterile, wide – trapped.

Eye-tracking metrics

Color: cyan	*Vegetation/Built*	Vegetation in the outdoor environment vs. built elements.
	Light/Shadow	Daylight-illuminated elements vs. elements in shadow.
	Far/Close	Elements far from the workstation vs. close elements.
	Walls/Other	Building walls enclosing the outdoor space vs. other elements.
	Workstation/Environment	The overall outdoor environment vs. workstation.
